# An anatomically designed duodenal stent with physiological drainage: effective endoscopic rescue in persistent perforation

**DOI:** 10.1055/a-2648-7338

**Published:** 2025-08-20

**Authors:** Shao-wei Li, Xin-yu Fu, Jinbang Peng, Saiqin He, Binbin Gu, Xinli Mao, Li-ping Ye

**Affiliations:** 1 Department of Gastroenterology, Taizhou Hospital of Zhejiang Province affiliated to Wenzhou Medical University, Linhai, China; 2 Key Laboratory of Minimally Invasive Techniques & Rapid Rehabilitation of Digestive System Tumor of Zhejiang Province, Linhai, China; 3 Institute of Digestive Diseases, Taizhou Hospital of Zhejiang Province affiliated to Wenzhou Medical University, Linhai, China; 4 36674 Dalian Medical University, Dalian, China

A 70-year-old woman with a refractory duodenal ulcer developed a 1.5-cm perforation at the duodenal bulb following two failed attempts at endoscopic hemostasis ( Fig. 1 ). Placement of an over-the-scope clip for closure was unsuccessful, and resulted in sepsis.

**Fig. 1:**
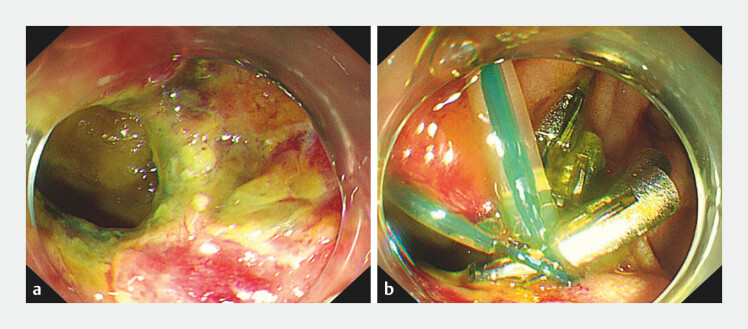
Endoscopic views showing: **a** the original duodenal perforation; **b** a failed closure attempt using over-the-scope clips.

A nitinol duodenal covered stent (22 mm in diameter, 10 cm in length) was deployed endoscopically ( Fig. 2 , Video 1 ). This stent integrates three key innovations: (i) a tripartite anchoring system – an umbrella-shaped gastric component for antral apposition, a cylindrical pyloric segment for radial fixation, and a bulbous duodenal anchor for coaptation; (ii) a papilla-aligned fenestration within the membranous mid-section to preserve physiological pancreatobiliary drainage; and (iii) optimized radial force distribution across segmented zones to minimize migration. Follow-up at 15 days confirmed closure of the complete perforation, and the stent was safely retrieved.

**Fig. 2:**
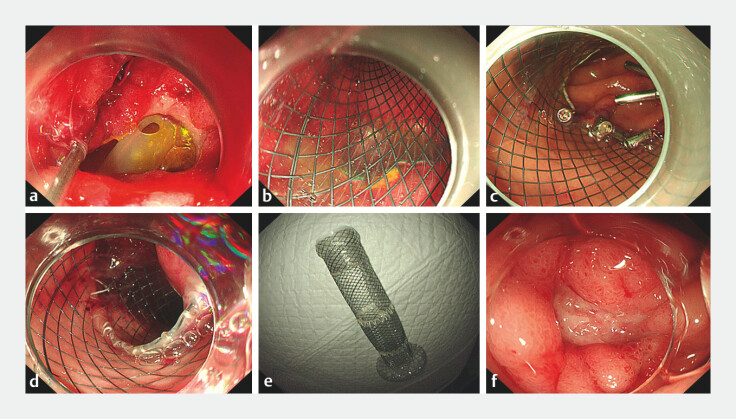
Images of treatment with a duodenal stent showing: **a** the fistula prior to insertion of the stent; **b** stent deployment; **c** the anchoring mechanism; **d** stent retrieval after the perforation had healed; **e** the anatomical design of the stent; **f** the healed perforation site.

An anatomically designed duodenal stent that can both obliterate the fistula and provide physiological drainage is used to close a perforation of a refractory duodenal ulcer in an elderly frail woman.Video 1

Duodenal perforations are challenging owing to anatomical fragility and the enzyme-rich secretions 1 2 . Conventional stents risk migration and obstructing drainage, thereby exacerbating leaks 3 . An anatomically designed duodenal stent uniquely addresses these limitations: its anchoring system ensures stable positioning without tissue injury, while the fenestration aligns precisely with the papilla, allowing continuous biliary/pancreatic flow into the bowel lumen. This design prevents reflux-related complications and promotes fistula healing by isolating the defect from digestive enzymes. The case underscores the stent’s dual role as a mechanical sealant and physiological conduit, which may be particularly critical in elderly patients with impaired healing.

Endoscopy_UCTN_Code_TTT_1AO_2AZ

## Notes

Endoscopy E-Videos https://eref.thieme.de/e-videos
*E-Videos* is an open access online section of the journal *Endoscopy* , reporting on interesting cases and new techniques in gastroenterological endoscopy. All papers include a high-quality video and are published with a Creative Commons CC-BY license. Endoscopy E-Videos qualify for HINARI discounts and waivers and eligibility is automatically checked during the submission process. We grant 100% waivers to articles whose corresponding authors are based in Group A countries and 50% waivers to those who are based in Group B countries as classified by Research4Life (see: https://www.research4life.org/access/eligibility/ ). This section has its own submission website at https://mc.manuscriptcentral.com/e-videos .
